# Prediction Value of KREBS Von Den Lungen-6 (KL-6) Biomarker in COVID-19 Patients: A Systematic Review and Meta-Analysis

**DOI:** 10.3390/jcm11216600

**Published:** 2022-11-07

**Authors:** Michal Matuszewski, Lukasz Szarpak, Zubaid Rafique, Frank W. Peacock, Michal Pruc, Piotr Szwed, Francesco Chirico, Alla Navolokina, Jerzy R. Ladny, Andrea Denegri

**Affiliations:** 1 Department of Anaesthesiology and Intensive Therapy at the Central Clinical Hospital of the Ministry of Interior and Administration, 02-507 Warsaw, Poland; 2 Henry JN Taub Department of Emergency Medicine, Baylor College of Medicine, Houston, TX 77030, USA; 3 Institute of Outcomes Research, Maria Sklodowska-Curie Medical Academy, 00-136 Warsaw, Poland; 4 Research Unit, Polish Society of Disaster Medicine, 05-806 Warsaw, Poland; 5 1st Chair and Department of Cardiology, Medical University of Warsaw, 02-097 Warsaw, Poland; 6 Post-Graduate School of Occupational Health, Università Cattolica del Sacro Cuore, 00168 Rome, Italy; 7 Health Service Department, Italian State Police, Ministry of the Interior, 20121 Milan, Italy; 8 Department of Public Health and Social Medicine, International European University, 03187 Kyiv, Ukraine; 9 Department of Emergency Medicine, Medical University of Bialystok, 15-089 Bialystok, Poland; 10 Cardiology Division, Department of Biomedical, Metabolic and Neural Sciences, University of Modena and Reggio Emilia, Policlinico di Modena, 41121 Modena, Italy

**Keywords:** biomarker, COVID-19, Krebs von den Lungen-6, SARS-CoV-2, severity

## Abstract

The SARS-CoV-2 (COVID-19) pandemic is a major issue that necessitates the use of cutting-edge disease prediction models. The aim of the study was to assess the existing evidence regarding association between Krebs von den Lungen-6 levels and COVID-19 severity. A literature search was performed on Web of Science, PubMed, Scopus and Cochrane Central Register of Controlled Trials databases from 1 January 2020 up to 2 August 2022. The electronic database search was supplemented by searching Google Scholar. In addition, reference lists of relative articles were also reviewed. KL-6 levels among COVID-19 positive vs. negative patients varied and amounted to 443.37 ± 249.33 vs. 205.73 ± 86.8 U/mL (MD = 275.33; 95%CI: 144.57 to 406.09; *p* < 0.001). The KL-6 level was 402.82 ± 261.16 U/mL in the severe group and was statistically significantly higher than in the non-severe group (297.38 ± 90.46 U/mL; MD = 192.45; 95%CI: 118.19 to 266.72; *p* < 0.001). The KL-6 level in the mild group was 272.28 ± 95.42 U/mL, compared to 268.04 ± 55.04 U/mL in the moderate COVID-19 group (MD = −12.58; 95%CI: −21.59 to −3.57; *p* = 0.006). Our meta-analysis indicates a significant association between increased KL-6 levels and SARS-CoV-2 infection. Moreover, KL-6 levels are significantly higher in patients with a more severe course of COVID-19, indicating that KL-6 may be a useful predictor to identify patients at risk for severe COVID-19.

## 1. Introduction

The coronavirus disease 2019 (COVID-19) pandemic, caused by the severe acute respiratory syndrome coronavirus 2 (SARS-CoV-2), has contributed to millions of deaths worldwide since its outbreak [1]. Despite vaccines, new antiviral drugs, greater treatment experience, and survival-favorable virus mutations, the pandemic is not completely controlled as of yet, and continues to threaten humans’ lives [2,3]. Moreover, there is still a risk of overloading health care systems due to the increased infectivity of new variants of the virus [3]. This, in turn, may contribute to increased mortality from causes other than COVID-19. Symptoms of disease caused by SARS-CoV-2 vary significantly from only fever and cough, to acute respiratory distress syndrome (ARDS), and often change dynamically within the progression of the disease. Hence, it is necessary to identify biomarkers that can stratify patients into cohorts of those who may develop severe diseases, versus populations who can be released from the hospital while still in the early stage of their disease. This is also important because of the possibility of offering intensified therapy with new drugs to patients at higher risk of severe COVID-19. Although biomarkers may enhance prognosis and outcomes, their high interpatient variability may have an impact on the investigations’ results. There are several biomarkers that may be used to evaluate the degree of COVID-19 infection. These markers may have a number of advantages, including the ability to recognize at-risk patients, stratify COVID-19 severity, help with the establishment of admission or intensive care criteria, provide treatment guidance through response assessment, evaluate prognosis, and frame ICU or regular ward discharge criteria.

The most commonly tested inflammatory biomarkers, including C-reactive protein (CRP), IL-6, and Procalcitonin (PCT), have proven insufficient in prospectively identifying patients who will suffer the severe course of COVID-19 [4]. However, combining several additional factors can increase their predictive value [5]. Additional prospective disease severity predictor candidates have been developed with other molecules, e.g., ferritin, lactate dehydrogenase, serum amyloid A or soluble interleukin-2-receptor (sIL2-R), but these have also proven mostly insufficient and not specific enough [6,7,8,9]. Finally, soluble urokinase-type plasminogen activator receptor (suPAR) is a promising predictor factor, but more data are needed to establish its potential clinical utility [10].

Krebs von den Lungen-6 (KL-6) is a high-molecular-weight glycoprotein that is released by type II alveolar pneumocytes and bronchial epithelial cells and has been shown to be a useful biomarker of alveolar epithelial proliferation and damage. KL-6 level has been reported to be increased in diseases such as acute respiratory distress syndrome (ARDS), pulmonary sarcoidosis, idiopathic interstitial pneumonia, hypersensitivity pneumonia, and collagen vascular disease-associated interstitial pneumonia. Moreover, KL-6 is associated with clinical outcomes and has been suggested for use evaluating disease activity [10,11,12]. Due to these properties, KL-6 has gained attention in COVID-19 evaluation as a molecule that may predict a more severe disease course.

In assessing the severity of COVID-19, computed tomography (CT) lung evaluation is of great value. The extent of lung involvement provides a more accurate assessment of disease severity than relying on somatic symptoms alone. Of note, the CT score correlates with serum KL-6 levels (*p* = 0.035) and was significantly higher in those with high KL-6 levels (>400 U/mL; 12.00, IQR 5.00–18.00, *p*-value 0.027). In addition, the KL-6 level was also significantly higher in COVID-19 positive subjects, compared to the negative group [10]. Interestingly, abnormal CT scans after 12 weeks from the onset of COVID-19 significantly correlated with elevated KL-6 levels upon admission [13].

Moreover, KL-6 has been positively correlated with CRP and IL-6 levels in patients with a severe course of COVID-19. The combination of these three prognostic factors differentiated severe from the mild-to-moderate disease [5]. An elevated KL-6 level on admission was an independent risk factor for prolonged hospitalization [14]. The above information indicates the value of baseline serum KL-6 level as a predictor of a more severe course of SARS-CoV-2 infection.

Conversely, some have reported no significant relationship between KL-6 levels and COVID-19 disease severity. KL-6 has also been demonstrated to be unrelated to persistent symptoms such as a feeling of shortness of breath 12 weeks after COVID-19 [13]. Given the inconsistencies in the data, our purpose was to perform a meta-analysis to summarize the information available in the literature on KL-6 and its utility to evaluate COVID-19 progression.

## 2. Materials and Methods

This systematic review and meta-analysis were prepared in accordance with the preferred reporting items for systematic reviews and meta-analyses (PRISMA) statement [15] and was registered with PROSPERO prior to completion of the initial search (registration No: CRD42022349526).

### 2.1. Search Strategy

Two reviewers (M.P. and A.N.) independently searched four major electronic databases (Web of Science, PubMed, Scopus, and Cochrane Central Register of Controlled Trials) from 1st, January 2020 up to 2nd, August 2022, to identify studies examining the prognostic value of Krebs von den Lungen-6 in COVID-19-hospitalized patients. The electronic database search was supplemented by searching Google Scholar. A specific and appropriate search strategy was used for each database. We used the following searching terms: “Krebs von den Lungen-6” OR “KL-6” AND “SARS-CoV-2” OR “COVID-19”. Search results were managed using EndNote software (version X7; Thomson Reuters). Additionally, reference lists of relative articles were also reviewed.

### 2.2. Study Selection

We included original studies that report the Krebs von den Lungen-6 levels among patients with COVID-19 on at least one or more of the following outcomes such as COVID-19 severity. Original articles available in English were included. The exclusion criteria for the meta-analysis were as follows: (1) studies involving data from pediatric patients; (2) case reports, editorial, conference papers, reviews; (3) studies published in other than English language; (4) studies lacking research indicators required for meta-analysis.

Two reviewers (L.S. and M.P.), independently and in duplicate, screened the titles and abstracts of the studies retrieved by the databases against the search criteria. Afterwards, the full texts of all potentially relevant articles were retrieved and independently assessed by the same reviewers. If any disagreement arose regarding the selection of literature papers disagreement was resolved through discussion with another reviewer (A.D.).

### 2.3. Data Extraction

Two investigators (L.S. and M.P.) performed study selection independently to select studies that met the above inclusion criteria. If potential disagreement arose, data extraction was resolved through discussion with another reviewer (A.D.). Data were collected using a predefined form. Data extracted included details regarding the publication data (i.e., first author name, year of publication, study design), population data (i.e., number of participants, age, male sex), KL-6 levels in predefined groups (COVID-19 positive and negative patients; mild and moderate COVID-19 severity groups; severe and non-severe COVID-19).

### 2.4. Quality and Risk of Bias Assessment

Two reviewers (M.P. and A.D.) independently assessed the individual studies for risk of bias. Any disagreements were also resolved by discussion with the third reviewer (L.S.). We used the Newcastle–Ottawa scale (NOS) to assess the methodological quality of observational studies with its design [16]. NOS score was categorized into three levels: low, moderate, and high quality, with the NOS scores of 0–5, 6–7, and 8–9. We performed funnel plot tests for asymmetry to investigate potential publication bias if there were more than 10 trials in a single meta-analysis.

### 2.5. Statistical Analysis

The meta-analysis was conducted in accordance with the Cochrane handbook. We analyze data using the STATA 14 software (StataCorp LP, College Station, TX, USA) and the RevMan 5.4 software (The Nordic Cochrane Center, The Cochrane Collaboration, 2014, Copenhagen, Denmark). For assess the KL-6 levels, we used mean differences (MDs) as the effect measure with 95% confidence intervals (CIs). In the case when KL-6 levels were reported as median with interquartile range, estimated means and standard deviations with the formula described by Hozo were used [17]. Heterogeneity was quantitatively assessed using Cochran’s Q statistics and Higgins’s index (I^2^), with 25%, 50%, and 75% considered moderate, substantial, and considerable heterogeneity, respectively [18]. The random-effects model was used for I^2^ > 50%; otherwise, the fixed effects model was employed. The Egger’s test was used to provide quantitative evidence. *p* < 0.05 was considered statistically significant.

## 3. Results

### 3.1. Study Characteristics

**Figure 1:**
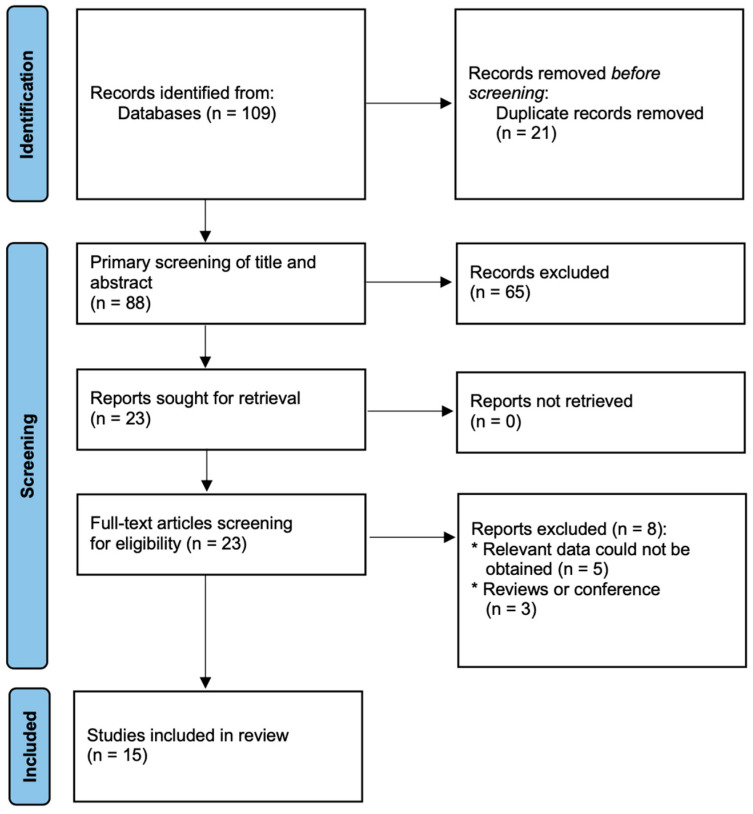
Flow chart of literature search and selection.

Our electronic literature search yielded 109 potentially relevant articles and one article was identified by hand searching. After elimination of duplicates, 88 records remained. Subsequent screening of titles and abstracts of the remaining records led to exclusion of 65 irrelevant records, leaving 23 articles. These articles were re-evaluated based on full-text contents, resulting in exclusion of 8 articles. Finally, 15 studies met the inclusion criteria and were included in our meta-analysis (Figure 1) [5,8,12,14,19,20,21,22,23,24,25,26,27,28,29]. All selected studies were published between 2020 and 2022. Detailed characteristics of the patients included in the meta-analysis is presented in Table 1.

Six studies reported KL-6 levels among COVID-19 positive vs. negative patients, eleven studies among severe vs. non-severe COVID-19 patients and three studies compared KL-6 levels between mild vs. moderate COVID-19 severity. Of the fifteen trials, six were performed in China, four in Japan, three in Italy, and one in each of the following countries: Belgium and Indonesia. The NOS scores of the eight included studies were ≥8 (Table 1).

### 3.2. KL-6 Meta-Analysis

**Figure 2:**
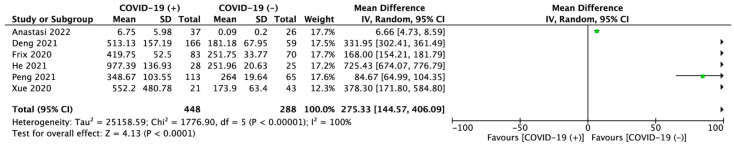
Forest plot of Krebs von den Lungen-6 levels (U/mL) among COVID-19 positive vs. negative patients. The center of each square represents the mean differences for individual trials, and the corresponding horizontal line stands for a 95% confidence interval. The diamonds represent pooled results [12,20,21,22,23,27].

Pooled analysis of KL-6 levels among COVID-19 positive vs. negative patients varied and amounted to 443.37 ± 249.33 vs. 205.73 ± 86.8 U/mL (MD = 275.33; 95%CI: 144.57 to 406.09; *p* < 0.001; Figure 2).

**Figure 3:**
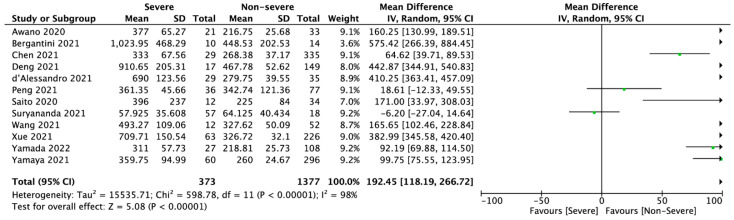
Forest plot of Krebs von den Lungen-6 levels (U/mL) among severe and non-severe COVID-19 patients. The center of each square represents the mean differences for individual trials, and the corresponding horizontal line stands for a 95% confidence interval. The diamonds represent pooled results [5,8,14,19,20,23,24,25,26,27,28,29].

12 studies reported KL-6 levels in severe and non-severe COVID-19 patients. Pooled analysis showed that KL-6 level was 402.82 ± 261.16 U/mL in severe group and was statistically significantly higher than in non-severe group (297.38 ± 90.46 U/mL; MD = 192.45; 95%CI: 118.19 to 266.72; *p* < 0.001; Figure 3).

**Figure 4:**
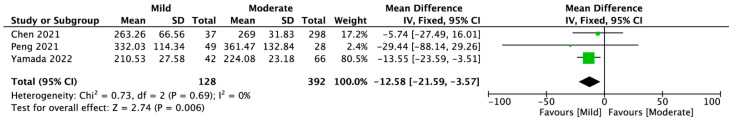
Forest plot of Krebs von den Lungen-6 levels (U/mL) among mild and moderate COVID-19 patient groups. The center of each square represents the mean differences for individual trials, and the corresponding horizontal line stands for a 95% confidence interval. The diamonds represent pooled results [14,23,28].

Three studies compere KL-6 marker among mild and moderate COVID-19 patient groups. KL-6 in mild group was 272.28 ± 95.42 U/mL, compared to 268.04 ± 55.04 U/mL in moderate COVID-19 group (MD = −12.58; 95%CI: −21.59 to −3.57; *p* = 0.006; Figure 4).

Sensitivity analysis based on the leave-one-out analysis showed that the pooled results were not influenced by a single trial. The above dependence applied to all comparisons included in the meta-analysis.

## 4. Discussion

We found that high levels of KL-6 are highly correlated with severe courses of COVID-19, and thus this marker may have the potential to be an excellent tool for the early identification of patients most likely to benefit from early antiviral therapy. Conversely, low KL-6 levels were associated with a mild disease course, and if validated, may be a useful tool in predicting a population who may be successfully managed in an outpatient environment.

The primary target of SARS-CoV-2 is definitely the lungs. Post-mortem studies have shown that the virus causes diffuse alveolar damage. In addition, COVID-19 has a higher incidence of thrombosis in the pulmonary vasculature compared to ARDS from other causes [30]. This is believed to be a function of the fact that SARS-CoV-2 uses the ACE2 receptor, and subsequently the Toll-like receptor, to invade pneumocytes and replicate its genome. Because KL-6 is a glycoprotein released by the type II alveolar pneumocytes and bronchial epithelial cells in various pulmonary diseases [30], the injury of pneumocytes and alveoli in COVID-19 may be pathophysiologically associated with elevated levels of KL-6 in the blood [31]. A disulfide link near the surface of the type II AECs’ epithelial cell membrane may be disrupted as a result of the inflammatory storm, and KL-6 can subsequently diffuse into the fluid and blood flow of the pulmonary epithelial lining [32]. It should be noted that because KL-6 is secreted by lung cells, as opposed to other inflammatory markers such as CRP, which are associated with broad inflammation, it has a substantial advantage when compared to other proinflammatory cytokines [33]. Therefore, KL-6 with the predictive value would predict who will be more likely to experience the fibrosing gradually, which can also be very helpful in assessing the COVID-19 patient’s condition, organizing the treatment for pulmonary fibrosis, or determining fibrosis following COVID-19 after the patient has been discharged from the hospital. This is crucial since 32–44.9% of individuals will develop lung fibrosis following COVID-19 [34,35]. Moreover, previous research has shown that the length of the illness plays a significant role in predicting the development of lung fibrosis following ARDS. About 4% of patients with diseases lasting less than a week, 24% of patients with diseases lasting between one and three weeks, and 61% of patients with diseases lasting longer than three weeks developed fibrosis [36].

Six studies in our meta-analysis compared KL-6 concentrations in COVID-19 cases and healthy subjects [12,20,21,22,23,27]. Results showed that KL-6 is significantly higher in COVID-19 than in healthy subjects. A previous meta-analysis evaluating KL-6 in COVID-19 positive and negative subjects also indicated significantly higher KL-6 levels in positive than in healthy subjects (standardized mean difference (SMD) = 1.34; 95%CI: 0.60 to 2.08) with high heterogeneity of data (*p* < 0.001, I^2^ = 93%) [22]. This indicates that SARS-CoV-2 infection causes an increase in KL-6, regardless of the symptoms caused, but the high heterogeneity of the data limits the usefulness of this information. Further studies are needed to obtain more homogeneous data.

Greater clinical value could be found in using KL-6 at admission to predict the subsequent course of COVID-19. Twelve studies involved in our meta-analysis assessed the KL-6 level according to the disease severity [6,8,14,19,20,23,24,25,26,27,28,29]. Our analysis demonstrated that there was a significantly higher level of KL-6 in patients suffering from severe COVID-19 than mild-to-moderate. Unfortunately, the heterogeneity of this data was high (I^2^ = 98%, *p* < 0.00001) decreasing the value of these findings. Nevertheless, our results are similar to those obtained in the previous meta-analyses. Ke et al., showed that serum KL-6 in patients with mild-to-moderate COVID-19 were significantly lower (SMD = −0.93; 95%CI: −1.22 to −0.65) than those in severe COVID-19 patients [37], although there was high heterogeneity of data. However, COVID-19 survivors had a significantly lower level of circulating KL-6 than non-survivors (SMD = −1.09; 95%CI: −1.63 to −0.55), and this analysis had low data heterogeneity (*p* = 0.52, I^2^ = 0%) [38]. Likewise, a low heterogeneity meta-analysis, performed by Naderi et al. showed that KL-6 was significantly higher in patients with severe than non-severe COVID-19 (SMD = 1.25; 95%CI: 0.99 to 1.5; *p* < 0.001) [39]. Another meta-analysis conducted by Witarto et al. presented similar results: that patients with severe COVID-19 had a higher level of KL-6 than those with the non-severe disease (SMD = 1.16; 95%CI = 0.69 to 1.63) [40]. In this study, heterogeneity was considered low (I^2^ < 25%). Taking the above results into account, it can be concluded that higher levels of KL-6 are associated with a more severe course of COVID-19, and the data in the literature are rather consistent. The problem of the studies that reduce the value of the obtained results is the high heterogeneity of the data. It may be due to the difficulty in defining the severe and non-severe course of the disease.

Our analysis also involved a comparison of KL-6 levels in mild and moderate COVID-19. Three articles contained the necessary data and were included in this analysis [14,23,29]. The results indicate, with low heterogeneity (I^2^ = 0%, *p* = 0.69), that mild COVID-19 is characterized by significantly lower KL-6 levels than moderate COVID-19. It illustrates that KL-6 levels increase with the severity of the disease, and further studies are needed that would determine the cutoff points for each degree of disease severity.

The main limitation of our study is the observational type of studies included in the meta-analysis. This results in a significant level of bias risk. Moreover, the analysis showed significant heterogeneity in the data, making it necessary to treat the obtained results with caution. Finally, there is always a risk of publication bias caused by a greater tendency to publish substantial results [41].

KL-6 has a rising clinical role in the research, with over 250 studies employing its clinical potential in the clinical trials registry alone [42]. The role of KL-6 use has already been confirmed in other lung diseases, such as pulmonary fibrosis, interstitial lung disease, idiopathic pulmonary fibrosis, diffuse parenchymal lung disease, and many others [43,44]. However, in the case of COVID-19, current research are not studies of KL-6 itself but already using it in the clinic to estimate patient’s lung function when testing new drugs or in patient care and clinical status. Detailed characteristics of studies using KL-6 for COVID-19 infection is presented in Table 2.

## 5. Conclusions

Our meta-analysis indicates a significant association between increased KL-6 levels and SARS-CoV-2 infection. Moreover, KL-6 levels are significantly higher in patients with a more severe course of COVID-19, indicating that KL-6 may be a useful predictor to identify patients at risk for severe COVID-19. However, the high heterogeneity of the data warrants cautions in interpreting these results.

## Figures and Tables

**Table 1:** A summary of study characteristics.

Study	Country	Study Group	No. of Participants	Age (Years)	Sex, Male (*n*, %)	NOS Score
Anastasi et al., 2022 [12]	Italy	COVID-19 (+)	37	66.5 ± 6.08	18 (48.6%)	8
		COVID-19 (−)	26	71.81 ± 7.27	10 (38.5%)	
Awano et al., 2020 [8]	Japan	Severe	21	65.5 ± 6.36	15 (71.4%)	9
		Non-severe	33	40.75 ± 4.91	23 (69.7%)	
Bergantini et al., 2021 [5]	Italy	Severe	10	65.2 ± 8	8 (80.0%)	8
		Non-severe	14	62.2 ± 15.6	11 (78.6%)	
		COVID-19 (−)	30	59 ± 9.8	18 (60.0%)	
Chen et al., 2021 [14]	China	Mild	37	NS	NS	8
		Moderate	298	NS	NS	
		Severe	29	NS	NS	
d’Alessandro et al., 2020 [19]	Italy	Severe	12	63 ± 2.34	9 (75.0%)	8
		Non-severe	10	60.75 ± 3.71	6 (60.0%)	
Deng et al., 2021 [20]	China	Severe	17	57.75 ± 4.35	9 (52.9%)	9
		Non-severe	149	48.13 ± 7.94	65 (43.6%)	
Frix et al., 2020 [21]	Belgium	COVID-19 (+)	83	71 ± 4	52 (62.6%)	8
		COVID-19 (−)	70	58 ± 3	35 (50.0%)	
He et al., 2021 [22]	China	COVID-19 (+)	28	64.56 ± 1.55	14 (50.0%)	8
		COVID-19 (−)	25	64.93 ± 1.63	16 (64.0%)	
Peng et al. 2021 [23]	China	Mild	49	44.5 ± 14	25 (51.0%)	9
		Moderate	28	51 ± 13.86	12 (42.9%)	
		Severe	36	56.5 ± 16.74	24 (66.7%)	
		COVID-19 (−)	65	47.75 ± 13.56	28 (43.1%)	
Saito et al., 2020 [24]	Japan	COVID-19 (+)	12	65.1 ± 10.7	7 (58.3%)	9
		COVID-19 (−)	34	49.6 ± 15.7	14 (41.2%)	
Suryananda et al., 2021 [25]	Indonesia	Severe	57	50.5 ± 13.85	38 (66.7%)	9
		Non-severe	18	49.75 ± 15.59	8 (44.4%)	
Wang et al., 2021 [26]	China	Severe	12	NS	NS	8
		Non-severe	52	NS	NS	
Xue et al., 2021 [27]	China	Severe	63	61.38 ± 4.19	31 (49.2%)	8
		Non-severe	226	54.75 ± 4.17	99 (43.8%)	
Yamada et al., 2022 [28]	Japan	Severe	27	64.25 ± 6.05	21 (77.8%)	8
		Non-severe	108	47 ± 12.85	48 (44.4%)	
Yamaya et al., 2021 [29]	Japan	Severe	60	NS	NS	8
		Non-severe	296	NS	NS	

Legend: NS: not specified.

**Table 2:** Currently ongoing research on KL-6 in the context of COVID-19 disease.

ClinicalTrial identifer	Study name	Status	Purpose of using KL-6	Time Frame
NCT04816760 [45]	Immune Cells Phenotypes During COVID-19 (IMMUNO-COVID)	Recruiting	Serum alveolar epithelial and endothelial cells biomarkers during SARS-CoV-2 infection incl. measurement of KL-6 using ELISA.	Day 0, Day 7, Day 14, Day 28
NCT05074875 [46]	COVID-19 Respiratory Outcomes Registry	Active, not recruiting	Examine the effects of COVID-19 on the presence of molecular biomarkers associated with Interstitial Lung Disease. Biomarkers prognostic for progression in PF patients incl. Krebs von den Lungen-6 (KL-6). Biomarkers elevated in PF (vs age-matched controls) incl. Krebs von den Lungen-6 (KL-6).	72 weeks
NCT04392531 [47]	Clinical Trial to Assess Efficacy of cyclosporine Plus Standard of Care in Hospitalized Patients with COVID-19	Completed- No Results Posted	Change in KL-6 change from baseline in KL-6 levels	Days 1, 8, 15, and end of study visit (14 days after discharge or 14 days after end of study treatment)
NCT04390061 [48]	TOFAcitinib Plus Hydroxycloroquine vs Hydroxycloroquine in Patients With COVID-19 Interstitial Pneumonia (TOFACoV-2)	Unknown	Identification of predictors of outcome. Role of some clinical and laboratory factors in predicting outcome incl. KL-6.	14 days
NCT04541680 [49]	Nintedanib for the Treatment of SARS-CoV-2 Induced Pulmonary Fibrosis (NINTECOR)	Recruiting	Compare change in lung injury, pulmonary hypertension, and inflammation biomarkers. Biomarker assay (KL-6, NT-proBNP, CRP, D-dimers)	At inclusion and 12 months

## Data Availability

The data that support the findings of this study are available on request from the corresponding author (L.S.).

## References

[1] Dzieciatkowski T., Szarpak L., Filipiak K.J., Jaguszewski M., Ladny J.R., Smereka J. (2020). COVID-19 challenge for modern medicine. Cardiol. J..

[2] Drożdżal S., Rosik J., Lechowicz K., Machaj F., Szostak B., Przybyciński J., Lorzadeh S., Kotfis K., Ghavami S., Łos M.J. (2021). An update on drugs with therapeutic potential for SARS-CoV-2 (COVID-19) treatment. Drug Resist. Updates.

[3] Nyberg T., Ferguson N.M., Nash S.G., Webster H.H., Flaxman S., Andrews N., Hinsley W., Bernal J.L., Kall M., Bhatt S. (2022). Comparative analysis of the risks of hospitalisation and death associated with SARS-CoV-2 omicron (B.1.1.529) and delta (B.1.617.2) variants in England: A cohort study. Lancet.

[4] Szarpak L., Nowak B., Kosior D., Zaczynski A., Filipiak K.J., Jaguszewski M.J. (2021). Cytokines as a predictor of COVID-19 severity: Evidence from meta-analysis. Pol. Arch. Intern. Med..

[5] Bergantini L., Bargagli E., D’Alessandro M., Refini R., Cameli P., Galasso L., Scapellato C., Montagnani F., Scolletta S., Franchi F. (2021). Prognostic bioindicators in severe COVID-19 patients. Cytokine.

[6] Fialek B., Pruc M., Smereka J., Jas R., Rahnama-Hezavah M., Denegri A., Szarpak A., Jaguszewski M.J., Peacock F.W., Szarpak L. (2022). Diagnostic value of lactate dehydrogenase in COVID-19: A systematic review and meta-analysis. Cardiol. J..

[7] Fialek B., Yanvarova O., Pruc M., Gasecka A., Skrobucha A., Boszko M., Ducki C., Cyran M., Szarpak L. (2022). Systematic review and meta-analysis of serum amyloid a prognostic value in patients with COVID-19. Disaster Emerg. Med. J..

[8] Awano N., Inomata M., Kuse N., Tone M., Takada K., Muto Y., Fujimoto K., Akagi Y., Mawatari M., Ueda A. (2020). Serum KL-6 level is a useful biomarker for evaluating the severity of coronavirus disease 2019. Respir. Investig..

[9] Szarpak L., Ruetzler K., Safiejko K., Hampel M., Pruc M., Koda L.K., Filipiak K.J., Jaguszewski M.J. (2021). Lactate dehydrogenase level as a COVID-19 severity marker. Am. J. Emerg. Med..

[10] Napolitano F., Di Spigna G., Vargas M., Iacovazzo C., Pinchera B., Cernia D.S., Ricciardone M., Covelli B., Servillo G., Gentile I. (2021). Soluble Urokinase Receptor as a Promising Marker for Early Prediction of Outcome in COVID-19 Hospitalized Patients. J. Clin. Med..

[11] Ishikawa N., Hattori N., Yokoyama A., Kohno N. (2012). Utility of KL-6/MUC1 in the clinical management of interstitial lung diseases. Respir. Investig..

[12] Anastasi E., Manganaro L., Guiducci E., Ciaglia S., Dolciami M., Spagnoli A., Alessandri F., Angeloni A., Vestri A., Catalano C. (2022). Association of serum Krebs von den Lungen-6 and chest CT as potential prognostic factors in severe acute respiratory syndrome SARS-CoV-2: A preliminary experience. Radiol. Med..

[13] Arnold D.T., Donald C., Lyon M., Hamilton F.W., Morley A.J., Attwood M., Dipper A., Barratt S.L. (2021). Krebs von den Lungen 6 (KL-6) as a marker for disease severity and persistent radiological abnormalities following COVID-19 infection at 12 weeks. PLoS ONE.

[14] Chen H., Qin R., Huang Z., Luo W., Zheng P., Huang H., Hu H., Wang H., Sun B. (2021). Clinical relevance of serum Krebs von den Lungen-6 levels in patients with coronavirus disease 2019. Cytokine.

[15] Page M.J., McKenzie J.E., Bossuyt P.M., Boutron I., Hoffmann T.C., Mulrow C.D., Shamseer L., Tetzlaff J.M., Akl E.A., Brennan S.E. (2021). The PRISMA 2020 statement: An updated guideline for reporting systematic reviews. BMJ.

[16] Stang A. (2010). Critical evaluation of the Newcastle-Ottawa scale for the assessment of the quality of nonrandomized studies in meta-analyses. Eur. J. Epidemiol..

[17] Hozo S.P., Djulbegovic B., Hozo I. (2005). Estimating the mean and variance from the median, range, and the size of a sample. BMC Med. Res. Methodol..

[18] Higgins J.P.T., Thompson S.G., Deeks J.J., Altman D.G. (2003). Measuring inconsistency in meta-analyses. BMJ.

[19] d’Alessandro M., Cameli P., Refini R.M., Bergantini L., Alonzi V., Lanzarone N., Bennett D., Rana G.D., Montagnani F., Scolletta S. (2020). Serum KL-6 concentrations as a novel biomarker of severe COVID-19. J. Med. Virol..

[20] Deng K., Fan Q., Yang Y., Deng X., He R., Tan Y., Lan Y., Deng X., Pan Y., Wang Y. (2021). Prognostic roles of KL-6 in disease severity and lung injury in COVID-19 patients: A longitudinal retrospective analysis. J. Med. Virol..

[21] Frix A.N., Schoneveld L., Ladang A., Henket M., Duysinx B., Vaillant F., Misset B., Moutschen M., Louis R., Cavalier E. (2020). Could KL-6 levels in COVID-19 help to predict lung disease?. Respir. Res..

[22] He L., Lu L., Zong M., Zhou H., Wang L., Chen N.Z., Yuan J.Y., Jiang E.P., Zheng L., Li Q. (2021). The Signicance of KL-6 as Prognosis Monitoring Biomarker in Patients with Severe COVID-19 From Stabilized Stage Toward Convalescence. Res. Sq..

[23] Peng D.-H., Luo Y., Huang L.-J., Liao F.-L., Liu Y.-Y., Tang P., Hu H.-N., Chen W. (2021). Correlation of Krebs von den Lungen-6 and fibronectin with pulmonary fibrosis in coronavirus disease 2019. Clin. Chim. Acta.

[24] Saito A., Kuronuma K., Moniwa K., Takahashi S., Takahashi H., Chiba H. (2020). Serum surfactant protein A and D may be novel biomarkers of COVID-19 pneumonia severity. Res. Sq..

[25] Suryananda T.D., Yudhawati R. (2021). Association of serum KL-6 levels on COVID-19 severity: A cross-sectional study design with purposive sampling. Ann. Med. Surg..

[26] Wang H., Chen L., Zhang Y., Liu L., Xu M., Gao Y., Li M. (2021). Detection of serum KL-6 and SARS-CoV-2 antibody in patients with coronavirus disease 2019 and the diagnostic value in severe disease. Res. Sq..

[27] Xue M., Zhang T., Chen H., Zeng Y., Lin R., Zhen Y., Li N., Huang Z., Hu H., Zhou L. (2021). Krebs Von den Lungen-6 as a predictive indicator for the risk of secondary pulmonary fibrosis and its reversibility in COVID-19 patients. Int. J. Biol. Sci..

[28] Yamada H., Okamoto M., Nagasaki Y., Yoshio S., Nouno T., Yano C., Tanaka T., Watanabe F., Shibata N., Arimizu Y. (2022). Analysis of Early Biomarkers Associated with the Development of Critical Respiratory Failure in Coronavirus Disease 2019 (COVID-19). Diagnostics.

[29] Yamaya T., Hagiwara E., Baba T., Kitayama T., Murohashi K., Higa K., Sato Y., Otoshi R., Tabata E., Shintani R. (2021). Serum Krebs von den Lungen-6 levels are associated with mortality and severity in patients with coronavirus disease 2019. Respir. Investig..

[30] Menter T., Haslbauer J.D., Nienhold R., Savic S., Hopfer H., Deigendesch N., Frank S., Turek D., Willi N., Pargger H. (2020). Post-mortem examination of COVID19 patients reveals diffuse alveolar damage with severe capillary congestion and variegated findings of lungs and other organs suggesting vascular dysfunction. Histopathology.

[31] Batah S.S., Fabro A.T. (2021). Pulmonary pathology of ARDS in COVID-19: A pathological review for clinicians. Respir. Med..

[32] Inoue Y., Barker E., Daniloff E., Kohno N., Hiwada K., Newman L.S. (1997). Pulmonary Epithelial Cell Injury and Alveolar–Capillary Permeability in Berylliosis. Am. J. Respir. Crit. Care Med..

[33] Sproston N.R., Ashworth J.J. (2018). Role of C-Reactive Protein at Sites of Inflammation and Infection. Front Immunol.

[34] Lee J.H., Yim J.-J., Park J. (2022). Pulmonary function and chest computed tomography abnormalities 6–12 months after recovery from COVID-19: A systematic review and meta-analysis. Respir. Res..

[35] Amin B.J.H., Kakamad F.H., Ahmed G.S., Ahmed S.F., Abdulla B.A., Mohammed S.H., Mikael T.M., Salih R.Q., Ali R.K., Salh A.M. (2022). Post COVID-19 pulmonary fibrosis; a meta-analysis study. Ann. Med. Surg..

[36] Rai D.K., Sharma P., Kumar R. (2021). Post covid 19 pulmonary fibrosis. Is it real threat?. Indian J. Tuberc..

[37] Xue M., Zheng P., Bian X., Huang Z., Huang H., Zeng Y., Hu H., Liu X., Zhou L., Sun B. (2020). Exploration and correlation analysis of changes in Krebs von den Lungen-6 levels in COVID-19 patients with different types in China. Biosci. Trends.

[38] Ke Y., Zhu Y., Chen S., Hu J., Chen R., Li W., Liu S. (2022). Clinical Utility of Circulating Pneumoproteins as Diagnostic and Prognostic Biomarkers in COVID-19: A Systematic Review and Meta-analysis. Res. Sq..

[39] Naderi N., Rahimzadeh M. (2022). Krebs von den Lungen-6 (KL-6) as a clinical marker for severe COVID-19: A systematic review and meta-analyses. Virology.

[40] Witarto A.P., Witarto B.S., Putra A.J.E., Pramudito S.L., Rosyid A.N. (2021). Serum Krebs von den Lungen-6 for Predicting the Severity of COVID-19 Lung Injury: A Systematic Review and Meta-Analysis. Iran. Biomed. J..

[41] Lin L., Chu H. (2018). Quantifying publication bias in meta-analysis. Biometrics.

[42] ClinicalTrials.gov. https://clinicaltrials.gov/ct2/results?cond=&term=KL-6+&cntry=&state=&city=&dist=.

[43] Kohno N., Ishikawa N., Deguchi N., Iwamoto H., Ohshimo S., Fujitaka K., Haruta Y., Murai H., Hattori N. (2012). KL-6 is a useful serum biomarker for early detection of interstitial lung disease. Eur. Respir. J..

[44] Zhang T., Shen P., Duan C., Gao L. (2021). KL-6 as an Immunological Biomarker Predicts the Severity, Progression, Acute Exacerbation, and Poor Outcomes of Interstitial Lung Disease: A Systematic Review and Meta-Analysis. Front. Immunol.

[45] ClinicalTrials.gov. https://clinicaltrials.gov/ct2/show/NCT04816760?term=KL-6+COVID-19&draw=2&rank=4.

[46] ClinicalTrials.gov. https://clinicaltrials.gov/ct2/show/NCT05074875?term=KL-6+COVID-19&draw=2&rank=5.

[47] ClinicalTrials.gov. https://clinicaltrials.gov/ct2/show/NCT04392531?term=KL-6+COVID-19&draw=2&rank=2.

[48] ClinicalTrials.gov. https://clinicaltrials.gov/ct2/show/NCT04390061?term=KL-6+COVID-19&draw=2&rank=3.

[49] ClinicalTrials.gov. https://clinicaltrials.gov/ct2/show/NCT04541680?term=KL-6+COVID-19&draw=2&rank=7.

